# Supplementary material for: Causal Relationship between Aging and Anorexia Nervosa: A White-Matter-Microstructure-Mediated Mendelian Randomization Analysis
Source: Biomedicines. 2024 Aug 16;12(8):1874. doi: 10.3390/biomedicines12081874 (PMC11351342; doi:10.3390/biomedicines12081874)
Supplement: Supplementary file 1 [file biomedicines-12-01874-s001.zip › Supplementary Figure/Figure S9/Leaveoneout plot of MR analysis for ubm-b-1939 to AN.pdf]

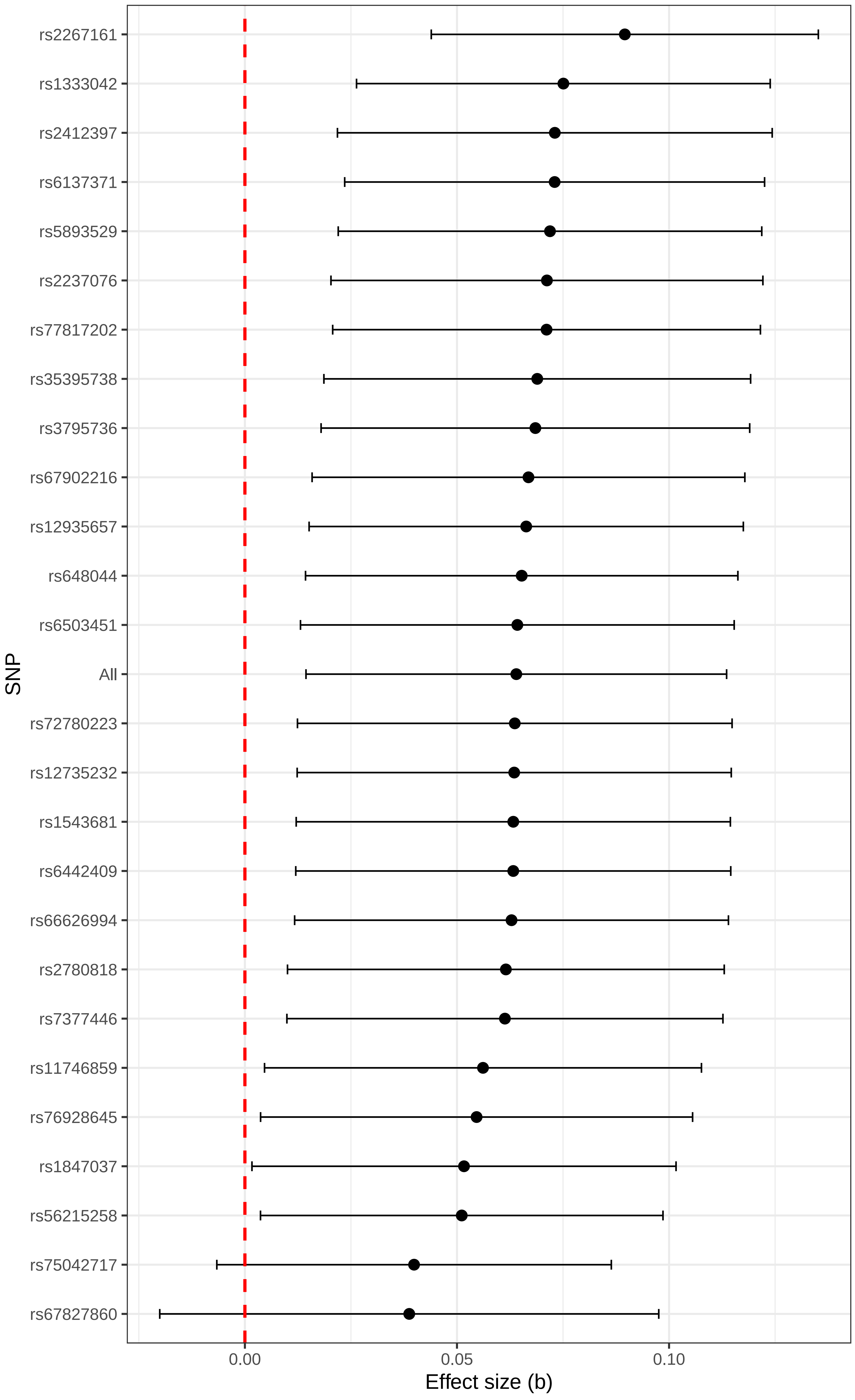
rs2267161
rs1333042
rs2412397
rs6137371
rs5893529
rs2237076
rs77817202
rs35395738
rs3795736
rs67902216
rs12935657
rs648044
rs6503451
All
rs72780223
rs12735232
rs1543681
rs6442409
rs66626994
rs2780818
rs7377446
rs11746859
rs76928645
rs1847037
rs56215258
rs75042717
rs67827860
SNP
0.00
0.05
0.10
Effect size (b)
